# Plant Defense against Herbivorous Pests: Exploiting Resistance and Tolerance Traits for Sustainable Crop Protection

**DOI:** 10.3389/fpls.2016.01132

**Published:** 2016-07-29

**Authors:** Carolyn Mitchell, Rex M. Brennan, Julie Graham, Alison J. Karley

**Affiliations:** ^1^Ecological Sciences, The James Hutton InstituteDundee, UK; ^2^Cell and Molecular Sciences, The James Hutton InstituteDundee, UK

**Keywords:** agro-ecosystem, arthropod, crop improvement, insect, natural enemy, trophic interactions

## Abstract

Interactions between plants and insect herbivores are important determinants of plant productivity in managed and natural vegetation. In response to attack, plants have evolved a range of defenses to reduce the threat of injury and loss of productivity. Crop losses from damage caused by arthropod pests can exceed 15% annually. Crop domestication and selection for improved yield and quality can alter the defensive capability of the crop, increasing reliance on artificial crop protection. Sustainable agriculture, however, depends on reduced chemical inputs. There is an urgent need, therefore, to identify plant defensive traits for crop improvement. Plant defense can be divided into resistance and tolerance strategies. Plant traits that confer herbivore resistance typically prevent or reduce herbivore damage through expression of traits that deter pests from settling, attaching to surfaces, feeding and reproducing, or that reduce palatability. Plant tolerance of herbivory involves expression of traits that limit the negative impact of herbivore damage on productivity and yield. Identifying the defensive traits expressed by plants to deter herbivores or limit herbivore damage, and understanding the underlying defense mechanisms, is crucial for crop scientists to exploit plant defensive traits in crop breeding. In this review, we assess the traits and mechanisms underpinning herbivore resistance and tolerance, and conclude that physical defense traits, plant vigor and herbivore-induced plant volatiles show considerable utility in pest control, along with mixed species crops. We highlight emerging approaches for accelerating the identification of plant defensive traits and facilitating their deployment to improve the future sustainability of crop protection.

## Introduction

Domestication of agricultural crops, estimated at 2500 species globally (Meyer et al., 2012), has involved artificial selection of desirable traits that enhance yield and quality of the harvested product. While breeding for agronomic targets in high input environments has successfully increased global crop productivity (Lynch, 2007), it has tended to produce modern crop varieties with relatively low levels of diversity (Khush, 2001). This reduced genetic diversity could limit the availability of varieties adapted for crop production under non-optimal conditions. Plant defensive traits can be lacking or expressed weakly in domesticated plants as a consequence of selection for other desirable traits (Chen et al., 2015). This poses a particular challenge for improving the sustainability of crop production as it suggests that modern varieties would perform poorly in low input systems with restricted pesticide use. While crop productivity has increased over the past century, combined global crop losses due to weeds, pests and diseases can be up to 40% (Oerke and Dehne, 2004). Across all vegetation systems, foliage, sap and root feeding herbivores remove >20% of net plant productivity (Agrawal, 2011). These losses occur despite increased pesticide use over recent decades (Oerke and Dehne, 2004), highlighting the need to develop sustainable approaches for pest control with less reliance on chemical inputs. To address concerns regarding human health, environmental safety and pesticide resistance, plant defensive traits could be exploited more widely in crop protection strategies.

Focusing on arthropod herbivores as pests, this review seeks, first, to summarize the plant defense strategies that have been documented in agricultural crops, second, to consider the potential utility of different types of crop defense, and, third, to highlight opportunities and technologies for improving the identification and deployment of plant defensive traits, particularly to achieve sustainable pest management under a changing environment.

## Plant Defense Strategies Toward Arthropod Pests

Plants have been successful in colonizing most environments and their success is due in part to their ability to resist or tolerate herbivore attack (Hanley et al., 2007). In a crop protection context, the system developed by Stout (2013) is particularly useful in differentiating between two plant defense strategies and the underpinning traits: resistance and tolerance. Resistance occurs when plant structural or chemical traits deter herbivore feeding and thus minimize the amount of herbivore damage experienced by the plant. Tolerance occurs when plant traits reduce the negative effects of herbivore damage on crop yield. This differentiation can allow defensive traits to be matched to the risk posed by the target pest: i.e., a high risk pest that should be reduced to low densities or eliminated vs. a low risk pest that can be tolerated within certain abundance thresholds. To identify suitable plant traits for crop protection against specific pests, we need a basic understanding of the mechanisms underpinning defensive traits, and how environmental conditions affect trait expression.

An important consideration is the extent to which defensive traits will provide durable pest control. Since plant resistance traits typically deter herbivore feeding, they are likely to impose a strong selection pressure on the herbivore to overcome plant resistance (Janzen, 1980). In contrast, plant tolerance traits are often assumed to have no effect on herbivore fitness, and therefore unlikely to impose selection on the herbivore (Strauss and Agrawal, 1999; Stowe et al., 2000). Stinchcombe (2002) challenges this assumption, suggesting that in some circumstances tolerance traits could influence herbivore performance, but few studies have investigated this possibility, particularly in a crop protection context. Either way, resistance traits are likely to impose a stronger selection pressure due to more severe impacts on pest fitness, suggesting that tolerance traits will be more stable (Weis and Franks, 2006) with greater chance of providing durable pest control.

## Resistance Traits and Mechanisms

The mechanism by which specific plant resistance traits deter herbivore feeding is likely to vary with the stage of insect establishment that they influence. Here, we summarize traits that are known to promote crop resistance to herbivores by (1) deterring pest landing, (2) preventing attachment and feeding, and (3) reducing plant palatability (**Table 1**).

**Table 1:** Examples of traits and underpinning mechanisms conferring crop resistance or tolerance to target arthropod pests.

Defense strategy	Mechanism	Trait and mode of action	Target pest	Crop host	Reference
**Resistance**	(1) Chemical deterrence of pest settling and feeding	Engineered elevated production of repellent alarm pheromone	*Myzus persicae*	*Triticum aestivum*	Bruce et al., 2015
		HIPV-induced attraction of maize stemborer parasitoids	*Chilo partellus*	*Cotesia sesamiae*	Tamiru et al., 2015
		Plant elicitor peptides induce plant defenses that impair Beet armyworm growth and attract its parasitoids	*Spodoptera exigua*	*Zea mays*	Huffaker et al., 2013
	
	(2) Physical barriers to pest attachment, feeding and oviposition	Epicuticular waxes differentially affect herbivore attachment	*Sitona lineatus*, *Acyrthosiphum pisum*	*Pisum sativum*	White and Eigenbrode, 2000
		Leaf surface waxes contribute to reduced performance of diamondback moth on cabbage	*Plutella xylostella*	*Brassica* sp.	Hariprasad and van Emden, 2010
		Glandular trichomes reduce mite movement	*Tetranychus urticae*	*Fragaria x ananassa*	Figueiredo et al., 2013
		Glandular trichomes reduce growth of corn earworm Non glandular trichomes impair Colorado potato beetle feeding and growth	*Helicoverpa zea* *Leptinotarsa decemlineata*	*Solanum lycopersicum*	Tian et al., 2012
		High density of non glandular trichomes prevent mite oviposition on raspberry	*Tetranychus urticae*	*Rubus idaeus*	Graham et al., 2014; Karley et al., 2016
	
	(3) Reduced plant palatability	Gramine alkaloid decreased aphid feeding, growth and survival	*Rhopalasiphum padi*	*Hordeum vulgare*	Zúñiga and Corcuera, 1986
		Benzoxazinoid synthesis decreased aphid growth and survival	*Rhopalasiphum padi*	*Zea mays*	Ahmad et al., 2011
		Aliphatic and indole glucosinolates reduced larval consumption and growth and slowed development on mature plants	*Mamestra brassicae* *Pieris rapae*	*Brassica oleracea* var. *acephala*	Santolamazza-Carbone et al., 2016
		Diterpenoid kauralexins deter feeding of corn borer larvae	*Ostrinia nubilalis*	*Zea mays*	Schmelz et al., 2011

**Tolerance**	(1) Photosynthesis and growth	Stimulate growth	*Amphorophora idaei*	*Rubus ideaus*	Johnson et al., 2012; Karley et al., 2016
		Increased root vigor	*Lepidiota stigma*	*Saccharum officinarum*	Allsop and Cox, 2002
	
	(2) Phenology	Delayed allocation to roots	*Diabrotica virgifera* *virgifera*	*Zea mays*	Robert et al., 2015

### Chemical Deterrence of Pest Settling and Feeding

Herbivore feeding and oviposition can induce plant defense, including emission of herbivore induced plant volatiles (HIPVs), which have been proposed as a new focus for crop pest resistance and biocontrol (Stenberg et al., 2015). Production of HIPVs signals herbivore presence that can attract natural enemies of the pest and even signal herbivore threat and induce defense responses in neighboring plants (e.g., Erb et al., 2015). A recent meta-analysis of HIPV studies (Rowen and Kaplan, 2016) concluded that domesticated plants tend to produce volatiles in larger quantities but of simpler composition compared to wild relatives (Chen et al., 2015; Rowen and Kaplan, 2016), suggesting that specific biosynthetic capabilities have been lost during crop breeding (Dicke, 2016). Wild relatives offer a genetic resource for reintroducing these traits into crops (Stenberg et al., 2015), and landraces can provide genetic variation in HIPV production and natural enemy attraction (e.g., parasitoids of maize stemborer: Tamiru et al., 2015). Engineering elevated volatile production into crop plants is feasible: for example, wheat plants modified to produce insect alarm pheromone both repelled aphids and attracted their natural enemies in controlled conditions, although this did not translate into improved aphid control in the field (Bruce et al., 2015).

‘Priming’ of plant defenses by cues that signal herbivore threat can allow rapid induction of plant defenses upon subsequent herbivore attack (Kim and Felton, 2013). Priming of inducible responses is an attractive proposition for crop breeding, allowing plant defense allocation to be balanced against the degree of herbivore pressure (Stenberg et al., 2015). The identity of plant elicitors and mechanisms of defense induction are emerging for several crop species (Huffaker et al., 2013; Huffaker, 2015), opening up opportunities for exploiting priming and defense induction traits in crop breeding (Stenberg et al., 2015).

### Physical Barriers

Plant structural traits (e.g., trichomes, spinescence, waxy cuticles, sclerophylly) can act as a physical barrier to arthropod pest attachment, feeding and oviposition; the plant cuticle and trichome density are two traits of particular focus in crop protection. Epicuticular waxes form a slippery film or crystals that prevent pests from attaching to the plant surface (White and Eigenbrode, 2000), ovipositing or feeding (Hariprasad and van Emden, 2010). Trichomes can prevent pest attachment and limit pest movement on crops (e.g., Tian et al., 2012; Figueiredo et al., 2013). While the effect of glandular trichomes is likely to have a chemical basis (see Reduced Plant Palatability, below), non-glandular trichomes act as a physical deterrent: oviposition by the generalist phytophagous mite, *Tetranychus uticae*, was significantly reduced on raspberry genotypes with high leaf trichome densities (Karley et al., 2016), and with identification of underlying genetic markers, this trait has potential utility in breeding for mite control (Graham et al., 2014). Trichomes can also have indirect negative (Michalska, 2003) and positive effects (Dai et al., 2010) on the target pest through their impact on the behavior of herbivore natural enemies. For example, abundance of the predatory mite *Typhlodromus pyri* on grape was associated positively with the presence of leaf trichomes, while its prey, the European red mite, favored grape varieties with low trichome density (Loughner et al., 2008). Trichomes tend to be more effective against insects that are small relative to trichome size; additionally, trichomes tend to deter sap feeding or leaf chewing insects to a greater extent than those feeding within plant tissues (Hanley et al., 2007).

### Reduced Plant Palatability

Plant compounds that are toxic or impair gut function in arthropods, produced constitutively or induced by herbivore damage, can enhance crop resistance to pests; examples include alkaloids (Zúñiga and Corcuera, 1986), benzoxazinoids (Ahmad et al., 2011), glucosinolates (Santolamazza-Carbone et al., 2016), and terpenoids (Schmelz et al., 2011). Plant breeding has tended to select against high levels of defensive compounds (Chen et al., 2015) due to their detrimental effects on crop quality for consumption. Targeted expression of defensive compounds in non-harvested organs (e.g., gossypol in vegetative structures of cotton; Palle et al., 2013) might allow tissue-specific engineering of chemical resistance into crops, although indirect effects of plant quality on biocontrol by natural enemies should be tested (Ågren et al., 2012). Another intriguing avenue is through symbiosis between cereal grasses and *Epichloë* fungal endophytes, allowing crops to benefit from fungal production of insecticidal alkaloids (Simpson et al., 2014).

Many plants deposit granular minerals in tissues that deter insect attack and feeding. A well-known example is silica accumulation in grasses (up to 2-5% silica by mass: Massey et al., 2006), which is abrasive, damaging herbivore feeding structures, and reducing digestibility (Massey and Hartley, 2009). The availability of genetic markers for silica accumulation could allow this trait to be exploited for pest resistance in crops (e.g., in rice: Bryant et al., 2011).

## Tolerance Traits and Mechanisms

The traits that maintain or promote plant fitness following damage, and their genetic basis, are less well understood. Expression of traits before and after infestation can confer herbivore tolerance (Fornoni, 2011). Plant tolerance traits (**Table 1**) are classically grouped into those that alter (i) physiological processes such as photosynthetic activity and growth, (ii) phenology, and (iii) use of stored nutrients (Strauss and Agrawal, 1999; Stowe et al., 2000; Tiffin, 2000). We focus on the first two categories as there are few examples of using stored nutrient reserves as a tolerance strategy, although storage organs are important for plant recovery from damage and offer an effective strategy against unpredictable herbivore attack if there is no tradeoff with plant productivity (Strauss and Agrawal, 1999).

### Photosynthesis and Growth

In many plant species, partial defoliation leads to increased photosynthetic rate in the remaining plant tissues (Strauss and Agrawal, 1999; Retuerto et al., 2004), suggesting that compensatory photosynthesis is a common physiological response to leaf damage (Tiffin, 2000). However, increased photosynthetic activity is not a universal response to herbivory and does not always drive compensatory growth, possibly due to resource diversion into resistance traits (Tiffin, 2000). Herbivore identity can determine whether changes in photosynthetic rate and growth occur: for example, compensatory photosysthesis is induced by several insect herbivores of soybean and drybean, but not by Mexican bean beetle (Peterson et al., 1998). By contrast, aphid feeding on the perennial crop red raspberry frequently stimulates plant growth and influences nitrogen physiology (Johnson et al., 2012), which could reflect tolerance to aphid herbivory through increased plant vigor (Karley et al., 2016). Similarly in sugarcane, clonal variation in tolerance to root-feeding whitegrub correlated with increased plant vigor (Allsop and Cox, 2002). Plant vigor can provide tolerance to herbivory in a range of plant species (Price, 1991); higher abundance and fitness of many insect herbivore groups on vigorous host plants (Cornelissen et al., 2008) could reflect increased ability of vigorous plants to tolerate attack. Although plant vigor is likely to be controlled by multiple loci, quantitative trait loci (QTL) studies have identified genetic markers for vigor (e.g., root and shoot vigor in raspberry: Graham et al., 2011, 2014) that could be deployed in crop breeding.

Activation of dormant buds after removal or damage to flowering or vegetative meristems is a further type of compensatory growth mechanism that allows plants to recover from herbivore attack that could be exploited in crop species with multiple meristems (Tiffin, 2000). In some circumstances, growth overcompensation is observed, which might be an attractive trait for improving crop tolerance in fertile agricultural conditions (Pilson, 2000), although any impact on the quality of the harvested product would need to be assessed.

### Phenology

Delayed growth, flower and fruit production following herbivore damage could promote herbivore tolerance by postponing plant development until the threat of attack has passed (Tiffin, 2000). For example, delayed resource allocation to roots is thought to underpin tolerance of western corn rootworm in herbivore-tolerant maize (Robert et al., 2015). The utility of these traits will depend on whether delayed development has a negative impact on yield and quality if the delay leads to crop flowering, pollination or ripening during non-optimal conditions.

## Selecting Traits to Optimize Plant Defense: Opportunities and Challenges

**FIGURE 1:**
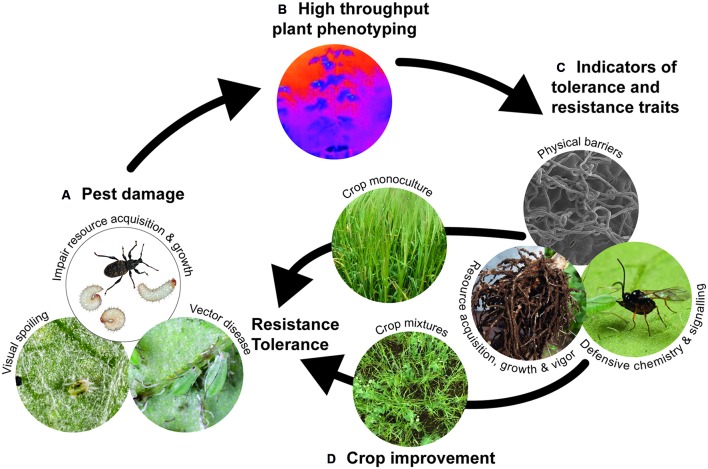
**Proposed strategy for improving crop protection against target arthropod pests. (A)** Identify the appropriate defense strategy (resistance or tolerance) depending on the type of damage and threat posed by the target pest; **(B)** develop high throughput phenotyping (HTP) technologies, particularly new imaging methods, for screening large plant populations to **(C)** identify appropriate indicators of resistance and tolerance traits; indicators could include reflectance properties that provide information about leaf surface characteristics and physical barriers, thermal and absorption data that provides information about stomatal conductance and water status, and therefore indicate photosynthetic activity and plant vigor, and absorption/reflectance data that characterizes leaf pigment composition and metabolic changes underpinning defense signaling (e.g., attracting natural enemies); **(D)** traditionally, desirable traits are characterized in germplasm monocultures, but phenotyping traits for use in crop mixtures is a potential route for durable pest control, particularly under environmental change.

Matching defensive traits to herbivore types to optimize pest control will depend on the nature of damage inflicted by the pest, whether direct feeding damage, removal of resources, visual spoiling or vectoring plant disease (**Figure 1**). Resistance traits are more desirable for maintaining disease vectors below threshold infestation densities. Tolerance traits are likely to be useful against non-vector pests that typically cause damage by removing resources and reducing plant growth (**Figure 1A**), although this has to be balanced against the possibility of pest spillover to neighboring crops or between cropping cycles. An important consideration is whether the target defensive trait has a negative impact on populations of beneficial organisms, particularly natural enemies of the pest. For example, while high trichome densities can reduce abundance of insect pests on cotton, trichomes can also impair the searching efficiency of herbivore natural enemies (Hagenbucher et al., 2013); by contrast, leafminers on tomato and their parasitoids are deterred by leaf trichomes, but trichomes and HIPVs have antagonistic effects on insect behavior (Wei et al., 2013). In some situations, incorporating plant traits that enhance natural enemy searching behavior might be more beneficial than enhancing pest resistance traits (Schmidt, 2014; Stenberg et al., 2015).

Technological advances in large-scale plant genotyping can accelerate selection of germplasm with desirable traits (Anderson and Mitchell-Olds, 2011), including herbivore defense. The rate-limiting step now resides in the ability to conduct high throughput phenotyping (HTP) to characterize desirable traits in large plant populations (**Figure 1B**). Imaging methodologies offer exciting opportunities for large-scale visualization of plant populations in controlled and field conditions, allowing semi-automated collection of light signals from the plant surface across a wide spectrum of wavelengths ranging between visible and infra-red (Fahlgren et al., 2015). Image-extracted traits provide information on canopy temperature, pigment composition and water status that can be linked to targeted measures of plant performance (Fahlgren et al., 2015). HTP approaches using imaging are already providing genetic markers for crop performance under abiotic stress (e.g., Prashar et al., 2013), and there is significant potential for applying imaging techniques to phenotype plant responses to insect pests (Goggin et al., 2015). For example, imaging methods could provide non-destructive indicators of physiological processes, such as stomatal conductance and water status, leaf pigment composition or photosynthetic activity, or plant vigor (**Figure 1C**) that indicate genotypic differences in ability to tolerate or resist insect pest attack above and belowground.

While studies of plant defensive traits frequently focus on a single trait and target pest, the underlying genetic control and expression of traits is likely to involve a suite of traits (Agrawal, 2011) expressed to defend against multiple pests above- and below-ground. Depending on the dominant crop pests, it might be feasible to focus on a single defensive trait, such as silica accumulation, which is effective against a range of herbivore types (Reynolds et al., 2009; Guntzer et al., 2012). Although there is surprisingly little evidence for trade-offs in plant investment between multiple defenses (Koricheva et al., 2004), understanding the genetic control of multiple traits remains a significant challenge for crop breeders. An alternative approach is to take advantage of defensive traits associated with different crop types grown as cultivar- or species-mixtures (**Figure 1D**). Plant diversification in crop systems often enhances natural enemy populations, suppresses arthropod pest populations and reduces crop damage (Letourneau et al., 2011) by providing a more complex habitat and heterogeneous resource for natural enemies, decreasing the density of preferred host plants, and interfering with host plant location and/or quality for herbivores (Jonsson et al., 2008; Letourneau et al., 2011). A good example of the latter effect is the negative impact of onions co-cropped with potato on attraction of potato aphids (Ninkovic et al., 2013). Increasing plant diversity in crop systems can confer additional benefits of yield stability and resource-use efficiency (Brooker et al., 2015). While there are many examples of the benefits of cultivating crop mixtures, particularly the ‘push-pull’ systems developed in sub-Saharan Africa for pest biocontrol (Pickett et al., 2014), there is significant opportunity for breeding crops with traits that optimize performance in mixtures (Ren et al., 2014).

## Conclusion and Future Perspectives

Crop domestication over recent decades has focused on plant traits that improve yield, enhance quality for human consumption and make the crop more amenable to existing cropping methods (Chen et al., 2015). Now, however, there is increasing focus on improving the sustainability of agriculture by reducing reliance on pesticides and other chemical inputs (War et al., 2012). From the studies highlighted here, there is considerable potential to exploit HIPVs, physical defenses and plant vigor to protect crops (and crop mixtures) against focal pests and to promote activity of natural enemies. A major uncertainty, however, is the durability of crop protection under a changing climate, which is anticipated to increase pest pressures on crops. Elevated temperatures are likely to accelerate insect development and increase the number of insect generations each season (DeLucia et al., 2012), elevated CO_2_ could decrease herbivore abundance but increase consumption (Stiling and Cornelissen, 2007), while intermittent water stress can enhance performance in certain herbivore guilds (Huberty and Denno, 2004). The effect of climate factors, individually or in concert, on expression of plant defense traits is uncertain. Elevated temperature and CO_2_ promote plant growth and volatile production, and can modulate defense signaling (DeLucia et al., 2012), which might strengthen expression of these tolerance/resistance traits. Conversely, these climate factors tend to reduce plant nutritional quality and decrease allocation to defensive compounds and physical structures, thus promoting plant consumption by herbivores (Stiling and Cornelissen, 2007; DeLucia et al., 2012), which suggests that crop protection from these physical and chemical resistance traits might be compromised under a changing climate. Applying imaging methods for HTP of target traits under conditions that mimic future climates (e.g., Rasmann et al., 2014), in parallel with optimizing crop defensive traits in mixtures, should assist crop scientists in identifying traits and trait combinations that are resilient to a changing environment, and that can be deployed as part of an integrated approach for sustainable crop protection.

## Author Contributions

The article was conceived by all authors, researched by CM and written by CM and AK, with corrections contributed by JG and RB.

## Conflict of Interest Statement

The authors declare that the research was conducted in the absence of any commercial or financial relationships that could be construed as a potential conflict of interest.
